# Incorporating a structural approach to reducing the burden of non-communicable diseases

**DOI:** 10.1186/s12992-018-0380-7

**Published:** 2018-07-06

**Authors:** Joshua S. Yang, Hadii M. Mamudu, Rijo John

**Affiliations:** 1 Department of Health Science, California State University, Fullerton, KHS 161A, 800 N. State College Blvd., Fullerton, CA 92834 USA; 2 College of Public Health, East Tennessee State University, Room G42-D, Lamb Hall, Johnson City, TN 37614 USA; 3 Centre for Public Policy Research, Anitha, 1st floor, S.A Road, Elamkulam, Kochi, Kerala 682020 India

**Keywords:** Non-communicable diseases (NCD), Global health, Public health policy, Social structure

## Abstract

**Background:**

Non-communicable diseases (NCDs) account for over two-thirds of deaths worldwide, and global efforts to address NCDs have accelerated. Current prevention and control efforts rely primarily on individual behavior/lifestyle approaches that place the onus of responsibility for health on the individual. These approaches, however, have not stopped the increasing trend of NCDs worldwide. Thus, there is urgent need for exploring alternative approaches in order to attain the aim of reducing global premature NCDs mortality by 25% by 2025, and meeting the NCD reduction objective in the Sustainable Development Goals.

**Discussion:**

We suggest the need for a structural approach to addressing the NCDs epidemic that integrates social science and public health theories. We evaluate two overarching principles (empowerment and human rights) and three social determinants of health (labor and employment, trade and industry, and macroeconomics) addressed in the 2013 Global Action Plan for the Prevention and Control of NCDs to demonstrate how a structural approach to NCDs can be incorporated into existing NCD interventions. For each area considered, theoretical considerations for structural thinking are provided and conclude with recommended actions.

**Conclusion:**

Achieving the global health agenda goals of reducing NCDs mortality will require a shift to a paradigm that embraces concerted efforts to address both behavioral/lifestyle factors and structural dimensions of NCDs.

## Background

Non-communicable diseases (NCDs) contribute to nearly 70% of all global deaths, about two-thirds of which occur in low- and middle-income countries (LMICs) [1, 2]. Without any intervention, total healthcare costs of NCDs in LMICs alone are expected to be over $7 trillion for the next 20 years [3]. Four groups of diseases – cardiovascular diseases (CVD), cancer, respiratory diseases, and diabetes – which are attributed to four major modifiable risk factors (tobacco use, physical inactivity, the harmful use of alcohol, and unhealthy diet) account for about 82% of NCDs-related deaths worldwide [4, 5].

In response to the growing burden of NCDs, the global community has worked through the World Health Organization (WHO) and the United Nations (UN) to reduce premature mortality due to NCDs by 25% by 2025 [4]. Years of concerted action to address NCDs culminated in the 2011 UN High Level Meeting on the Prevention and Control of Non-communicable Diseases (HLM) [6], a watershed moment in global health governance by bringing global awareness to an issue that had been overshadowed by traditional issues in international health such as infectious diseases. The resulting UN Political Declaration on NCDs in 2011 (NCDs Declaration) [7] outlined global priorities and commitments for NCDs, which were subsequently operationalized in three major institutional agreements under the auspices of the WHO: the Global Monitoring Framework (GMF) that created 9 voluntary global targets and 25 indicators [8], Global Action Plan (GAP) that outlined a set of cost-effective interventions for Member States to consider implementing [8], and Global Coordinating Mechanism (GCM) designed to coordinate global action on NCDs [9]. Significantly, NCDs have been incorporated into the Sustainable Development Goals (SDGs) [10], making NCDs not only a health but also a development issue.

In 2014, the UN Secretary General noted that, in spite of the developments described above, progress toward achieving the benchmarks in the GMF “has been insufficient and highly uneven” ([11], p. 1); slow progress has been attributed to insufficient funding, weak health systems, poor public understanding of NCDs, lack of political prioritization, slow civil society engagement, inappropriate framing of NCDs, legitimacy challenges, management of industrial stakeholders, and the complexity of tackling NCDs [12–16]. In 2017, the UN Secretary General reported that implementation of time-bound commitments are below benchmarks set in 2014, citing political choices, weak health systems, limited national capacity, insufficient financing, and industry interference as factors impeding progress [17]. With preparation for the third HLM in September 2018 underway, we suggest that in addition to these factors, the specific recommendations promoted by the WHO and UN to reduce the burden of NCDs – particularly those outlined in the GAP – should be critically reexamined to assess whether they are sufficient to achieve global NCDs prevention and control goals. We propose that countries should go beyond individual behavior/lifestyle approaches favored in the GAP to understand and address structural underpinnings of NCDs. In invoking social structure, we suggest that a new avenue of research and policy action is needed to address enduring social relations and systems that determine the distribution of NCDs and their risk factors among different subpopulations. An emphasis on individual behavior/lifestyle avoids the fundamental sources of NCDs; thus consideration of social, political, and economic systems and their impact on NCDs is needed.

## Individual versus structural approach

Critical review of the NCDs Declaration and subsequent documents suggests that the recommendations for action have been highly individualized, with limited emphasis on structural issues such as the social determinants of NCDs [18, 19]. Though the importance of social determinants of NCDs is acknowledged, these reviews indicate that specific recommendations for action provided by guiding documents tend to undergo a “lifestyle drift,” shifting attention from social determinants of health and other structural issues to changing individual behavior/lifestyle [19].

This focus on the individual as the locus of change is consistent with trends in public health practice since the mid-twentieth century. Along with the emerging dominance of the biomedical model of health, changes in disease burden have resulted in practical and theoretical approaches to public health that focus on individual change; hence, the emphasis on social-psychological theories such as the Health Belief Model and Social Cognitive Theory [20]. Whether this focus on individual behavior/lifestyle modification is referred to as behavioralism, individualism, medicalization, patient-centered or a lifestyle approach, the end result is the same: shifting the locus of control to the individual with diminished emphasis on social determinants of NCDs, the effects of social structural factors, and the responsibility or duty of governmental actors to alleviate NCD burden. An individualized frame of NCDs urges individuals to cease tobacco use, eat a healthy diet, reduce alcohol consumption, and engage in physical activity. As such, the central responsibility of the government is to engineer incentives and disincentives for certain behaviors through policies such as taxes and create the exchange “markets” within which individuals make decisions to improve their health status. Increasingly, however, calls to incorporate structural factors into the NCDs discourse have emerged [21, 22].

Less well developed within the NCD literature is theorizing and recommendations for action which focus on change within social, political, and economic systems. The social sciences, however, provide rich theoretical and practical insights into the structure of society that may hasten progress towards global NCD goals. What we describe as a *structural* approach to NCDs focuses on enduring social arrangements that determine the pattern and distribution of NCDs and their risk factors in a society. Structuralism views society as a complex system with interlocking parts that work together to ensure social stability, but is independent of the action of individuals. Sociologist Emile Durkheim viewed society as more than a collection of individuals and that conditions external to the individual determine an individual’s actions [23], and Talcott Parsons asserted that individual action is rooted in societal norms and constrained by societal values [24]. Thus, even if we assume that individuals have the ability to make rational choice with respect to healthy behavior/lifestyle, such a choice occurs within certain boundaries set by society, government, and organizations [25]. We suggest that a structural approach conceives of the NCD epidemic as the byproduct of changes to domestic and international systems that have dramatically changed modes of living and created environments that encourage the adoption of harmful patterns of behavior. These structural changes have been facilitated by several factors, including industrialization, urbanization, globalization, expansion of capitalism, and rapidly changing technology.

We do not view social systems as completely deterministic of individual and social action with respect to NCDs. In response to Objective 5 of the GAP which calls for greater research on the macroeconomic and social determinants of health, it is our intention in this paper to heighten understanding of how to make structural thinking an integral part of the current NCDs discourse for a more inclusive, coherent, and efficacious NCDs research and policy agenda. Doing so embeds human agency within real and powerful social systems that facilitate some social action while constraining others, and can lead to more effective efforts to reduce NCD mortality. While several structural issues co-exist at different levels of governance, we selected from the GAP two overarching principles (empowerment and human rights) and three social determinants (labor and employment, trade and industry, and macroeconomics) as examples of how conceptual and theoretical considerations in structural thinking may inform recommended actions that address structural factors related to NCDs.

## An alternative approach to NCDs: Social structure

### Power and empowerment

#### Theoretical considerations

Power is a major driver of health that can be used to represent the interests of groups vulnerable to and affected by NCDs in shaping decision making related to NCDs [26, 27]. To this effect, the GAP holds as one of its overarching principles “empowering of people and communities” ([4], p. 3), with civil society playing a key role in empowering society. Member States are thus urged to create national NCDs governance frameworks that engage and empower a wide range of stakeholders to catalyze “societal change and shape a systematic society-wide national response to address [NCDs], their social, environmental, and economic determinants” ([4], p. 24).

The inclusion and empowerment of a broadly construed notion of people and communities is essential. The particular approach adopted, however, is limited to what Lukes [28] labels a one-dimensional view of power. This approach focuses on actors increasing their capacity to influence others in creating NCD policies and programs and not reforming systems of power. As Lukes suggests, power has other manifestations. A two-dimensional view includes “mobilization of bias” which allows some actors to control how decisions are made, limiting the ability of other actors to have their grievances and interests attended to [29]. Lukes proposes a third dimension of power, when a group shapes “perceptions, cognitions and preferences in such a way that [others] accept their role in the existing order of things” (p. 28). Shiffman [30] has a similar typology of power for global health, calling attention to (a) structural power, the ways in which people are organized in relation to one another; and (b) productive power, the creation of meaning and frameworks through which people come to understand the world.

Empowerment of people and communities alone does not sufficiently address power relations among stakeholders [29]. This is particularly important for NCDs because of the central role of economic actors such as transnational corporations (TNCs) and unhealthy commodities industries with the resources and expertise to shape perceptions and preferences that ultimately lead to NCDs [28]. Member States must legitimize the experiences and views of traditionally excluded or underrepresented groups, and reform systems of power that currently protect vested and entrenched interests. States can reform power structures by changing sociocultural practices, economic relations, institutional rules and norms on participation, agenda setting, and decision making in national and global arenas.

Rushton and Williams [31] suggest that the productive power of neoliberalism and its proponents has established it as a “deep core” of global health governance, the standard by which arguments for action on global health are legitimized and prioritized. The result is a reliance on privatized, individualized, and market-based solutions which crowd out other orientations toward NCDs and preserve systems and protect vested interests that produce NCDs. Systems of power must be reassessed and reconfigured in order to derive the full benefit of incorporating new actors into efforts to prevent and control NCDs.

#### Recommended actions

Reconfiguring relationships between powerful and entrenched commercial interests and populations at risk for or suffering from NCDs requires first a reconsideration of assumptions that governmental engagement and “co-regulation” with the private sector is the preferred model of governance for NCDs [14]. The question of private sector engagement in NCDs governance has been taken up by Working Group 3 of the WHO GCM, the international body charged with advancing implementation of the WHO GAP [32]. The WHO GCM Working Group 3 established governments as having the primary responsibility for responding to NCDs and recommends they establish “statutory and regulatory frameworks to enable more concrete contributions from the diverse range of private sector entities to NCD prevention and control goals and targets” ([33], p. 10). It also asserts, however, that contributions from the private sector for NCD prevention and control need to be scaled up and emphasizes the positive role of various sectors of private industry while deemphasizing “reservations about engaging or collaborating with some private sector entities in tackling NCDs” ([33], p. 8). The WHO GCM Working Group 3 exercises productive power in implicitly establishing co-accountability, conditioned on management of conflicts of interest, as the dominant model of NCD governance. There are at least two shortcomings with this view. First, it obscures the primary issue in private sector engagement: the role of unhealthy commodity industries. As the major drivers of NCDs, greater policy emphasis should be placed on the need for public regulation and market intervention of unhealthy commodity industries [34]. Second, partnering with and adopting strategies of private industry for NCD prevention and control reinforces the “neoliberal” deep core in global health and reliance on market mechanisms. Therefore, the first step in altering power relations in NCDs governance is to cultivate the productive power of other stakeholders to develop and legitimize alternative approaches. These include human rights (see below), justice, ethics, health equity, and other frames. Academic institutions and civil society groups in particular have an important role to play in establishing and advocating for an alternative basis for NCD governance.

Changes to power structures help ensure that empowerment of communities leads to more than token participation with little ability to affect decision making. For example, one model of accountability places primary responsibility on national NCD commissions in which civil society and academia play a part [35]. An alternative accountability model would create formal mechanisms embedded throughout NCD activities by which governments and private industry are directly accountable to the public and obligated to enact remedies needed to reduce the burden of NCDs [36]. Increasing government and private sector accountability to the public and obligating them to enact remedies are initial steps to changing institutional rules, agenda setting authority and decision making processes which shift power from government and industry to the public.

### Human rights

#### Theoretical considerations

Human rights is a paradigm that can transform global action on NCDs. The recognition that every person has the fundamental right to the enjoyment of the highest attainable standard of health is urged in the GAP. Human rights, however, are not given further attention, leaving open to interpretation what is meant by adopting a “human rights approach.”

Consistent with the right to health under the UN Universal Declaration of Human Rights (Article 25) [37] and other human rights treaties and conventions, a human rights approach is a potentially fruitful frame with which to respond to global NCDs [38]. Although a rights – defined as justified claims that impose duties or responsibilities on others – approach may be an area of synergy with NCDs, there is a lack of attention linking human rights and NCDs. A human rights approach to NCDs should address a wide array of issues, including: the centrality of health as a human right compared to political and civil rights; who has claims on rights related to NCDs; the nature and scope of the claims made; who guarantors of rights ought to be; conflict among rights; and limiting of rights claims due to their societal cost.

At a most basic level, a human rights-based approach to NCDs requires three components: parties who claim rights, parties that have a duty to fulfill rights claims, and something that is claimed or can be adjudicated. While rights holders could be conceived of as those who suffer from or are exposed to risk factors for developing NCDs, identifying duty-bearers and those liable for NCDs burden becomes complex due to the multidimensional nature of NCDs. Understanding rights claims as positive (a claim to something, such as health services or healthier food options) and negative (a claim of protection from something, such as predatory marketing) suggests that the governments and industry have particularly large roles to play as duty bearers of rights claims.

Taken together with changes in relations of power described above, integration of a human rights approach into NCDs prevention and control efforts can transform moral claims to political and legal claims. State responsibility to respect, protect, and fulfill human rights [39] can be legitimized, and guarantors of those rights held to greater account. The GAP provides a menu of policy options for governments to consider, measures to *provide* health education and promotion, universal health care, alternatives for healthier diet and physical activity, and active transport; and to *protect* from secondhand smoke exposure, marketing of unhealthy food to children and of alcoholic beverages, and exposure to unhealthy food and beverages and tobacco products. A similar transformation of moral to political and legal human rights claims on TNCs is more challenging; TNCs are obligated first and foremost to their fiduciary responsibility to corporate investors. Thus, the state role as protector of human rights against third party violations is essential, requiring greater state responsiveness to rights claims made by people and communities who are vulnerable to developing NCDs vis-à-vis corporate interests. This can be brought about only if power is more evenly distributed among stakeholders. Thus, a structural approach conceives of the right to health as something that can be claimed against governments, industry, and other duty-bearers for the development of and investment in NCD prevention and control policies and programs.

#### Recommended actions

Human rights language in the GAP and other NCD policy documents are cursory, limiting their utility [40]. Synergies between NCDs and the global human rights system exist, however, with additional linkages possible within the current global human rights architecture. In order to fully utilize the international human rights machinery such as the UN Special Rapporteur for Human Rights, an NCD human rights framework – including processes, goals, and indicators – should be developed and disseminated to WHO Member States and civil society organizations as an analog to the WHO GAP. The development of an NCD human rights framework should be a funding priority of foundations working in the NCD domain to spur action by civil society groups and academic institutions. At the national and sub-national levels, judicial systems will need to be reformed and strengthened to recognize and enforce human rights claims even when doing so impinges on other parts of government or the private sector [41]. Such systemic change will only occur through international and domestic pressure. Expanded legal expertise and assistance to navigate local and national legislative and legal systems will be needed to support advocates and the public in having their human rights claims adjudicated in accordance with international human rights obligations.

Changes in power relationships and wider adoption of human rights as a guiding principle in policy and law provide an opportunity for specific policy changes which can help prevent and control NCDs. We provide examples of a structural approach to NCDs prevention and control for three sectors: labor and employment, trade and industry, and macroeconomics. Specific recommendations for action in each sector focus on actions within states, and not on global governance, reflecting the centrality of Member States as having primary responsibility for NCD prevention and control in WHO and UN documents.

### Labor and employment

#### Theoretical considerations

Work and employment make up essential elements of daily experience in environments recognized for their effect on NCDs through social relationships and exposure to or protection from health hazards. Normative workplace attitudes and behavior toward tobacco and unhealthy alcohol use may have a strong effect on development of NCDs. For example, a culture of tobacco use may be seen in some low wage occupations and unlikely to be covered by smoke-free workplace policies. On the other hand, tobacco and unhealthy alcohol use may be a part of the culture of white collar workers where informal interactions may center around tobacco and alcohol use [42, 43].

The effects of work and employment on NCDs, however, extend beyond the work environment. The structure of the labor market – including wages, precariousness of work, opportunities for fair and decent employment, and work-life balance [44, 45] – influence vulnerability to NCDs. Low wages, precarious work, and few opportunities for fair and decent employment can lead to irregular and inconsistent work hours and the need for multiple jobs. This in turn hampers one’s ability to engage in active transport, recreation, and preparation of nutritious meals.

Urbanization, the availability of employment, required work hours for a living wage, and labor instability also affect NCDs by modifying class-based exposure to NCDs risk factors. While urbanization can affect exposure to shared risk factors for NCDs [1], precarious employment and underemployment may lead to poorer health outcomes through behavioral coping such as tobacco and unhealthy alcohol use, unhealthy dietary choices, and sedentary lifestyles [45]. Among the world’s poorest billion – those excluded from the formal labor system – NCDs have a different epidemiologic pattern but may make up one third of overall disease burden [46]. This suggests that material poverty, and not behavioral risks, is likely the driving cause of NCDs for the world’s bottom billion.

#### Recommended actions

A labor systems approach to NCDs includes more than mitigating occupational exposures to NCDs risk factors. It targets power relations which govern work and the very nature and structure of work and employment [47]. Policy recommendations should transform work to mitigate NCDs through financial security, social mobility, and personal development and fulfillment. Specific policy goals include full employment, a healthy living wage, appropriate preparation and training for work, increased employee involvement over the control and nature of work, and a strong social safety net for transitions between employments. The Employment Conditions Knowledge Network (EMCONET) [47] has detailed specific policies which countries should explore to reduce potential negative effects of labor and employment markets on NCDs. Recommendations include altering power relations through unionization and collective bargaining, and reshaping the nature of labor through measures such as universal access to public education; legislating a living minimum wage; avoiding wage discrimination; income redistribution through progressive taxation; promoting full employment and working time flexibility; regulating downsizing to prevent job insecurity and outsourcing; and strengthening and expanding occupational health and safety standards.

### Trade and industry

#### Theoretical considerations

As Moodie et al. [34] have noted, TNCs are major drivers of NCDs. The tobacco, alcohol, and ultra-processed food industries not only manufacture and market products that promote NCDs, but they also engage in a variety of strategies that determine the preferences of individuals and undermine public health policies and programs. As a result, they call for greater public regulation of unhealthy commodity industries through taxation, pricing, product bans, and restrictions on advertising and sponsorship. The GAP has included a number of regulatory measures, most strongly for tobacco control, as policy options for Member States to consider in their national efforts. In addition, consistent with the Article 5.3 of the WHO Framework Convention on Tobacco Control (WHO FCTC) [48] and the NCD Declaration [7], the GAP calls for exclusion of the tobacco industry in developing health promotion interventions.

The GAP, however, does not address more fundamental threats to states’ ability to protect public health in light of the growing position of TNCs in the international political economic system, especially with expanding globalization and international trade. The 1648 Treaty of Westphalia created states as political and sovereign entities and the central actors in international relations [49]. State sovereignty, however, has undergone tremendous transformation through the proliferation of TNCs [50].

The relationship between states and TNCs has undergone different changes since the General Agreement on Tariffs and Trade (GATT) emerged in the early 1950s to set the foundation for the contemporary multilateral trading system [51] currently governed by the World Trade Organization (WTO). Of concern is whether the emergence of TNCs on the world stage and as key actors in international relations has undermined the power of governments to protect the health of populations within its jurisdiction [44, 52]. TNCs are strongly supported by the dominant liberal international economy, which makes it difficult for states to regulate their activities. Multilateral trade, along with a plethora of regional and bilateral investment treaties, appears to favor trade more than health and provides TNCs with leverage to challenge national health policies [53]. Thus, venues such as the WTO’s Dispute Settlement Body (DSB), the World Bank’s International Center for the Settlement of Investment Disputes (ICSID) and the UN Commission on International Trade Law (UNCITRAL) have been used by both states and TNCs to challenge states’ effort to protect the health of their populations. For example, the tobacco industry has used these venues to challenge the ability of states to enact and implement tobacco control policies consistent with the provisions of the WHO FCTC [48].

#### Recommended actions

The Investor-State Dispute Settlement (ISDS) mechanism is a provision in new era trade agreements that highlights the tension between national sovereignty and interests of TNCs [54–56]. ISDS provisions allow corporate investors to bring claims directly against governments outside of existing WTO structures to seek compensation when governmental actions are viewed as harming foreign investment. Thus, the adoption of ISDS mechanisms in trade agreements may discourage or actively undermine public health policies through “regulatory chill,” including policies designed to safeguard the public from the products manufactured by unhealthy commodity industries. In response, Member States should be encouraged to exclude ISDS provisions in trade agreements and other measures that may erode the ability of governments to enact NCD prevention and control policies in favor of TNCs’ interests [54]. Doing so is not without precedent. Brazil’s Cooperation and Facilitation Investment Agreements model limits dispute settlements to state-state relations [57]. New Zealand has side agreements with five countries in the Trans-Pacific Partnership excluding compulsory ISDS processes [58]. In addition, countries should make all trade negotiations transparent; require health and human rights impact assessments as part of trade agreements; prevent trade agreements from interfering or undermining governments’ ability to regulate and protect products related to NCDs; ensure that the WHO FCTC is recognized as the guiding standard for tobacco-related disputes; and prevent barriers to affordable medicines [54]. In doing this, it is critical to distinguish between industries whose activities produce “global public good” from those that produce “global public bad.”

### Macroeconomics

#### Theoretical considerations

Increasing NCDs prevalence can cause social dislocation by pushing many people out of work, leading to an increase in the ratio of dependent population and negatively impacting the macro-economy. As the number of economically productive people decrease, taxable household income declines which, on the one hand, limits the public sector’s ability to expand investments while on the other hand diminishes resources diverted to addressing NCDs.

The dominant view of the linkages between economics and NCDs is to focus on improving health as a driver of economic growth. Studies have shown that a healthy population is an engine for economic growth [59]. From 2000 to 2011, for example, about 24% of the full income growth in LMICs resulted from health improvements alone and reduction in mortality accounted for about 11% of the economic growth in these countries [60]. Healthy societies typically have a competitive advantage that fuels productivity resulting in higher economic growth [61]. Globally, it is estimated that leaving the four main NCDs and mental health conditions unaddressed could cost the world $47 trillion in lost economic output between 2010 and 2030 [62]. For LMICs, the cumulative economic losses from NCDs are estimated to surpass $7 trillion over the period 2011–2025. On a per-person basis, the annual losses amount to $25 in LMICs, $50 in lower middle-income countries and $139 in upper middle-income countries [62]. In contrast, the cost of implementing a core set of NCDs intervention strategies, including population-based measures for reducing tobacco use, harmful alcohol use, unhealthy diet, and physical inactivity, are estimated at $2 billion per year for all LMICs, which is less than $0.40 per person [63].

A structural perspective expands the view of the linkage between economic policies and NCDs to include the economic system and targets changes in macroeconomic policy and socioeconomic structures of societies to improve population health. Income inequality, which has been shown to negatively affect health [64, 65], is an example of how economic systems structure experiences of NCDs. Emphasis on economic development and health has obscured the need to address distributional effects of growth policies. Inequality is an inherent element of the capitalist system yet has detrimental effects not only for health but other social and political indicators as well [66]. Thus, income and wealth redistribution policies and programs are important considerations in reduction of NCDs. Saez [67] asserts that from World War II to the 1970s, redistributional and regulatory policies reduced inequality without slowing economic growth suggesting that population health can be improved by reducing inequality through redistribution policies while still fostering economic growth. Thus, careful examination of potential policy and programmatic tools aimed at reducing inequality through structural changes to economic systems should be encouraged for their potential positive effect on NCDs.

#### Recommended actions

Numerous growth policies that may reduce income inequality are available to policymakers [68]. Investments in education such as early childhood education, universal primary education, better teacher recruitment and training, support for at-risk students, and reducing discriminatory educational attitudes and policies can expand access to and improve the quality of education, increase human capital, and reduce labor income inequalities. Increasing tax revenue and the progressivity of taxes can also reduce inequality. Social policies which promote the integration of immigrants, improve labor market outcomes for women through empowerment and reducing gender disparities, and reduce discrimination in the job and housing markets can also reduce inequality and alleviate poverty. Additionally, since policies and programs of international governmental organizations such as the World Bank and nongovernmental organizations impact the economy of many countries, particularly LMICs, these organizations should consider aligning with civil society organizations in undertaking projects that enhance educational attainment or alleviate poverty.

## Conclusion

The third HLM on NCDs in 2018 will review progress on NCDs at the half way point between the NCD Declaration (2011) and 2025, the year by which governments have agreed to reduce NCD burden by 25%. In order to hasten the progress on NCDs, the UN Secretary General has called for a “paradigm shift” to “encourage more holistic approaches to [NCDs]” ([17], p. 21). We suggest a new paradigm for NCDs needs to incorporate a greater focus on underlying structural dimensions of NCDs with dominant behavioral approaches. Drawing on social science theories, we have presented five examples of how to apply structural thinking to two overarching principles (power and empowerment, human rights) and three policy domains (labor and employment, trade and industry, and macroeconomics) from the GAP. The goal of structural interventions in each of these areas is to identify and modify systems that facilitate or impede health-seeking behaviors, placing governments and society – not individuals – as the locus of change in efforts to address NCDs. The NCD policy, advocacy, programmatic, and research communities will need to articulate and advance a broader set of structural interventions across sectors to provide an expanded set of recommended actions for policymakers and invigorate social mobilization for NCDs.

Reorganizing power relationships to elevate the position of traditionally excluded or marginalized groups in governance of NCDs is fundamental to structural NCD interventions. Though civil society and academic institutions play an important role in developing alternative governance models to the co-regulation approach embraced in current governance approaches, it is the WHO and other UN agencies which will need to use their global position and productive power to legitimize and establish governance alternatives among Member States. New power structures that focus on increasing government and private sector accountability to the public and obligating them to enact remedies are an initial step to shift power from government and industry to the public. A human rights framework is one potential basis of an alternative governance model. In order for a more complete elaboration of a human rights model to be developed, such an effort will need to be a funding priority to spur action by civil society and academic institutions. Expanded legal expertise and assistance to navigate local and national legal systems will be needed to make judicial systems responsive to human rights claims against entrenched and powerful interests and support the public in having their human rights claims adjudicated in accordance with international standards.

Changes in power relationships and wider adoption of human rights as a guiding principle in policy and law provide an opportunity for specific policy changes in labor and employment, trade and industry, and macroeconomics. Fair employment and decent work should be the goals of structural changes to employment relations [69]. A global trade regime which protects against NCDs should exclude ISDS measures from all trade agreements; make all trade negotiations transparent; require health and human rights impact assessment as part of trade agreements; and prevent trade agreements from interfering, undermining, or preempting governments’ ability to regulate and protect products related to NCDs. Finally, macroeconomic policies that can minimize inequalities at the root of NCDs include investments in education, increasing tax revenue and progressivity of taxes, promoting integration of immigrants, improving labor market outcomes for women, and reducing discrimination in the job and housing markets.

A shift in paradigm will require courageous leadership at all levels of governance to challenge vested interests that benefit from the status quo and resist meaningful structural change. Preventing undue suffering and millions of deaths from NCDs requires nothing less.

## Abbreviations

CVD: Cardiovascular disease
DSB: Dispute settlement body
FCTC: Framework Convention on Tobacco Control
FDI: Foreign direct investment
GAP: Global action plan
GATT: General Agreement on Tariffs and Trade
GCM: Global coordinating mechanism
HLM: High level meeting
ICSID: International Center for the Settlement of Investment Disputes
ISDS: Investor-state dispute settlement
LMIC: Low- and middle-income country
NCD: Noncommunicable disease
R&D: Research & development
SDG: Sustainable development goal
SES: Socioeconomic status
TNC: Transnational corporation
UNCITRAL: UN Commission on International Trade Law
WHO: World Health Organization
